# Correcting motion induced fluorescence artifacts in two-channel neural imaging

**DOI:** 10.1371/journal.pcbi.1010421

**Published:** 2022-09-28

**Authors:** Matthew S. Creamer, Kevin S. Chen, Andrew M. Leifer, Jonathan W. Pillow

**Affiliations:** 1 Princeton Neuroscience Institute, Princeton University, Princeton, New Jersey, United States of America; 2 Department of Physics, Princeton University, Princeton, New Jersey, United States of America; 3 Department of Psychology, Princeton University, Princeton, New Jersey, United States of America; University of California Santa Barbara, UNITED STATES

## Abstract

Imaging neural activity in a behaving animal presents unique challenges in part because motion from an animal’s movement creates artifacts in fluorescence intensity time-series that are difficult to distinguish from neural signals of interest. One approach to mitigating these artifacts is to image two channels simultaneously: one that captures an activity-dependent fluorophore, such as GCaMP, and another that captures an activity-independent fluorophore such as RFP. Because the activity-independent channel contains the same motion artifacts as the activity-dependent channel, but no neural signals, the two together can be used to identify and remove the artifacts. However, existing approaches for this correction, such as taking the ratio of the two channels, do not account for channel-independent noise in the measured fluorescence. Here, we present Two-channel Motion Artifact Correction (TMAC), a method which seeks to remove artifacts by specifying a generative model of the two channel fluorescence that incorporates motion artifact, neural activity, and noise. We use Bayesian inference to infer latent neural activity under this model, thus reducing the motion artifact present in the measured fluorescence traces. We further present a novel method for evaluating ground-truth performance of motion correction algorithms by comparing the decodability of behavior from two types of neural recordings; a recording that had both an activity-dependent fluorophore and an activity-independent fluorophore (GCaMP and RFP) and a recording where both fluorophores were activity-independent (GFP and RFP). A successful motion correction method should decode behavior from the first type of recording, but not the second. We use this metric to systematically compare five models for removing motion artifacts from fluorescent time traces. We decode locomotion from a GCaMP expressing animal 20x more accurately on average than from control when using TMAC inferred activity and outperforms all other methods of motion correction tested, the best of which were ~8x more accurate than control.

## Author summary

Optical imaging of neural activity using fluorescent indicators is a powerful technique for studying the brain, yet despite the widespread success of this technique many difficulties remain. Imaging moving animals can be challenging, because the animal’s movements may introduce changes in fluorescence intensity that are unrelated to neural activity, known as motion artifacts. This work explores computational approaches to correct for these artifacts. In particular, we focus on the special case of two-channel imaging, where two indicators are present in the same cell: an activity-dependent indicator and an activity-independent indicator. We compare prior methods for motion artifact correction and present a new method, Two-channel Motion Artifact Correction (TMAC) method. TMAC uses a generative model of the fluorescence to infer the latent neural activity without motion artifacts. We demonstrate that TMAC outperforms alternative methods for motion correction when applied to experimental data from moving animals.

This is a *PLOS Computational Biology Methods* paper.

## Introduction

Population fluorescent imaging of calcium-sensitive fluorescent indicators is a powerful approach for recording cellular neural dynamics [1,2]. Calcium imaging’s widespread adoption has benefited from extensive development of computational algorithms to find and segment neurons of interest [3], to extract and denoise calcium signals [4,5], and to infer their underlying voltage signals [6]. An important goal of systems neuroscience is to probe the neural basis of animal behavior [7,8] and calcium imaging has been used to measure neural activity in awake and behaving animals [9–11]. However, animal motion during calcium imaging poses unique challenges both for segmenting and tracking neurons and for accurately extracting calcium traces. Many computational approaches have been proposed to register, segment and track neurons in the presence of motion [9,12–21]. These approaches account for the gross movement of a neuron relative to its neighbors or within the field of view. By contrast, there have been relatively few efforts to account and correct for motion-related changes to the extracted fluorescence intensity time-series itself. Accounting for these latter effects of motion, however, is particularly important because motion related changes to fluorescence intensity can appear similar to behavior-related neural signals of interest. Here, we construct a method called Two-channel Motion Artifact Correction (TMAC) which uses the fluorescence traces from two-channel imaging to correct for shared motion artifacts between the two channels.

**Fig 1:**
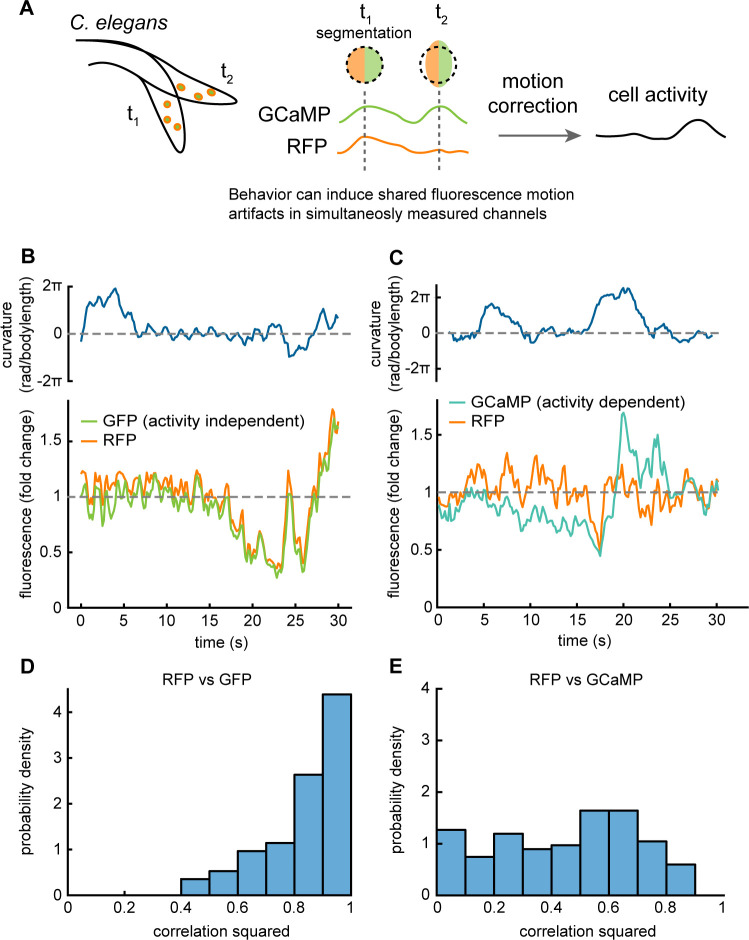
Behavior induces motion artifacts in optical recordings. A) Diagram of a moving *C*. *elegans* undergoing motion and deformation during simultaneous measurements of GCaMP and RFP fluorescence. Deformations of the cell can conspire with imperfect segmentation to create motion artifacts. RFP intensity fluctuations reflect motion or noise. GCaMP intensity fluctuations reflect motion, calcium activity, and noise. B) *Top*, Animal body curvature. *Bottom* fluorescence of a neuron from a whole-brain recording of a moving worm expressing both GFP and RFP. Both fluorophores are activity-independent, yet we observe large highly correlated fluctuations in the two-channel fluorescence. C) *Top*, Body curvature. *Bottom*, fluorescence of a single neuron taken from a whole-brain recording in a moving worm expressing both GCaMP and RFP. The two channels still show correlated fluctuations despite the activity dependence of GCaMP. D) Histogram of the Pearson correlation coefficient squared between the red and the green channel for a dataset of whole-brain recordings from 10 moving GFP, RFP control worms. E) Same as D but in 9 GCaMP, RFP worms.

When a neuron deforms or moves relative to an imaging plane, its fluorescence intensity can change unrelated to neural activity **(Fig 1A)**. While multiple factors could contribute to these motion induced changes, at least some of them arise from subtleties of image acquisition and segmentation. Even though the number of fluorescent molecules in a neuron is constant, the neuron’s shape, orientation and position are not. Accurately measuring fluorescence therefore requires carefully accounting for which voxels a neuron occupies, the intensity of each voxel, and the shape of the imaging hardware’s point-spread function used to calculate the voxel. For example, as a neuron changes its shape with respect to the point spread function, it may cover less voxels but be brighter in the voxels that remain. In real-world imaging conditions it can be challenging to detect subtle changes to the boundary of a neuron, especially in densely labeled neural populations, and similarly the point-spread function is not always sufficiently characterized. These challenges are particularly acute in recordings of moving animals, such as *C*. *elegans* [11,22,23], *Hydra* [24], *Drosophila* larvae [25,26], and zebrafish larvae [27,28] that all exhibit large head deformations during movement.

Because these artifacts arise from motion, they have the potential to confound interpretations of the neural correlates of behavior. For example, if a neuron appears to change fluorescence intensity during head bending, it may be hard to disambiguate whether that fluorescent transient reflects calcium activity or motion artifact. Two-channel imaging offers one strategy to account for motion-induced changes in fluorescence by measuring a calcium indicator like GCaMP in one channel and a calcium-independent indicator like RFP in another. Any intensity fluctuations of the calcium-independent channel must be either noise or motion artifact. In principle, this knowledge can be used to account for and correct artifacts in the calcium imaging channel. Crucially, whatever the source of these artifacts, as long as they affect the red and green channel equally, two-channel motion correction should be able to account for and remove them.

Two-channel calcium imaging has its origins in the use of FRET based calcium indicators that use a donor and receptor and reports calcium signals as the ratio between the two channels [29]. The ratiometric approach further proved useful in freely moving animals [10], because the ratio is less sensitive to motion-induced fluorescent changes common to both channels. Single-channel GCaMP based indicators have now surpassed FRET-based indicators in terms of popularity because of their speed and brightness [30]. However, the use of two-channel imaging has persisted in moving animals in part for its ability to remove motion artifacts with methods such as taking the ratio of the two channels [22,23,31–33], or by performing linear regression [11,34]. To date there has not been a systematic comparison of different mathematical approaches to account for motion induced changes to fluorescent intensity in two-channel imaging. TMAC explicitly models the statistical distributions of the noise, artifact, and cell activity and uses Bayesian inference to find activity uncontaminated by motion artifact. We use experimental data to compare TMAC with four other approaches to motion artifact removal and demonstrate that it outperforms previous methods.

## Results

### Motion artifacts

We inspected two-channel calcium imaging recordings of neurons in moving *C*. *elegans* from Hallinen et al., 2021 (**S1 Table**) for signs of motion-related calcium transients. We considered two types of recordings: those of control animals that express only the calcium-insensitive fluorophores GFP and RFP and those of calcium-sensitive animals that expressed the calcium indicator GCaMP in addition to RFP. In each case, GFP or GCaMP was imaged in the green channel and RFP was imaged in the red channel. In order to account for differences in intensity, we divide each channel by its time average and report fluorescence as fold change from the mean. In control animals, the green and red fluorescent intensity had large fluctuations relative to the mean (**Fig 1B)** and were highly correlated to each other (**Fig 1D**). Because these animals contained no neural-related signals and only motion, we conclude that motion artifacts contribute substantially to fluctuations in fluorescence. We also note the strong correlation between the GFP and RFP fluorescence, which suggests that the motion artifact is shared between the two channels. These shared fluctuations are what make motion correction using a second channel possible.

In GCaMP recordings, we also observed a correlation between the green and red channels (**Fig 1D and 1E)**, although less so than in control recordings. The presence of activity-related signal in the green channel likely explains this difference. The correlation that remains between the red and the green channel suggests that the red channel could be used to correct for shared motion artifacts in the green channel.

### Model

**Fig 2:**
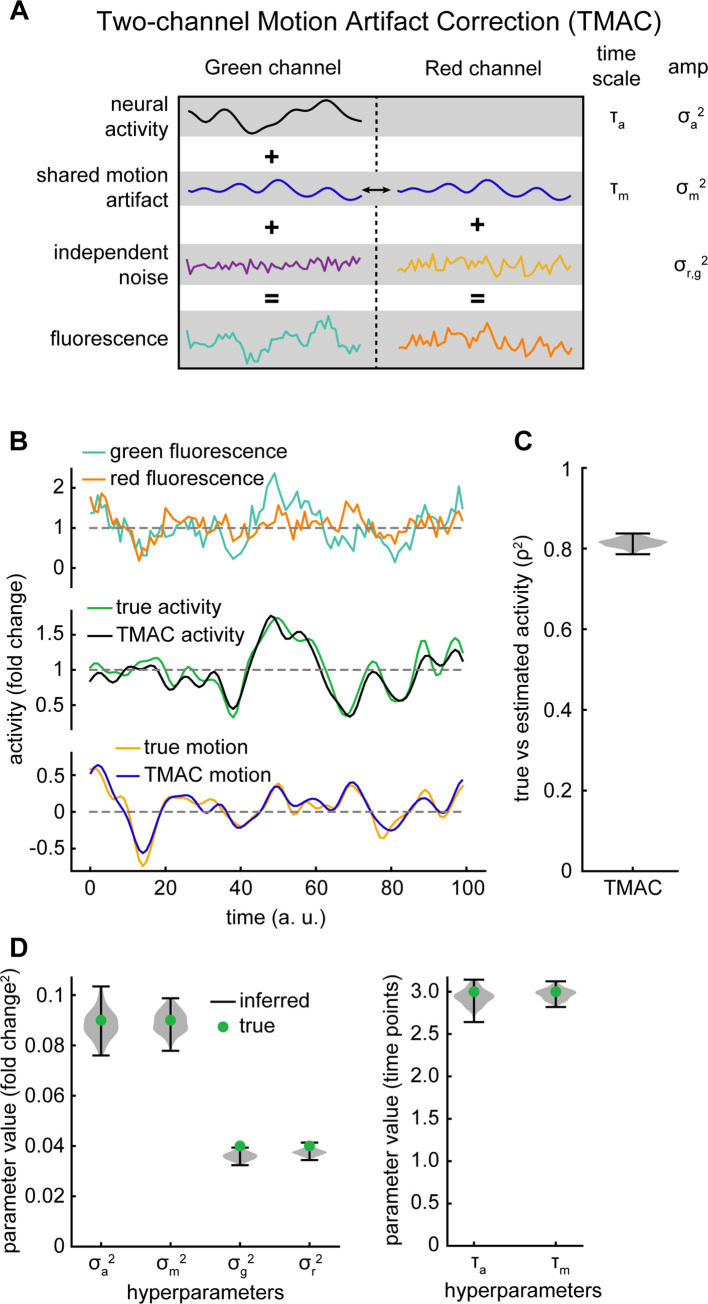
TMAC infers activity and hyperparameters from synthetic data. A) Diagram of the structure of TMAC. The green channel is modeled as the sum of the calcium activity, motion artifact, and independent gaussian noise. The red channel is modeled as the sum of the motion artifact and independent gaussian noise. The motion artifact is shared between the two channels. B) Top: fluorescence from a synthetic green and red channel. Middle and bottom: inferred activity and motion compared with the true activity and motion. C) Correlation squared between estimated activity from TMAC and true activity over many synthetic datasets. D) Violin plot of inferred and true parameter values when fitting TMAC.

To account for fluctuations in fluorescence induced by motion artifact, we propose a method called Two-channel Motion Artifact Correction (TMAC) (**Fig 2A**). This method relies on a latent variable model of calcium fluorescence, which we use to infer the latent neural activity of each neuron. Intuitively, inferring the latent activity from the model amounts to subtracting the motion signals present in the red channel from the green channel while accounting for channel independent noise. We first process the fluorescence data by dividing each channel by its time average, such that the processed data has mean 1 and units of fold change from the mean. By specifying our model fluorescence as fold change from the mean the parameters do not depend strongly on experimental conditions such as cell expression, laser intensity, or image acquisition time, allowing us to compare across different neurons and fluorophores (**Fig 1B and 1C**).

We define the fluorescence from a neural activity-dependent green channel and a neural activity-independent red channel as follows. Let ***r*** and ***g*** denote vectors of the preprocessed red channel and green channel fluorescence from the same neuron. We assume that ***r*** is the sum of a latent motion artifact time series ***m*** and additive Gaussian white noise ***ε***_***r***_. Similarly, we assume the green channel measurements ***g***, arise as the sum of the same latent motion artifact ***m***, a latent time series of neural activity ***a***, and Gaussian white noise ***ε***_***g***_. To obtain a complete generative model, we assume Gaussian process priors over ***m*** and ***a***, which specify that they evolve smoothly over time. Formally the model can be written as:

$$r=1+m+\epsilon_{r}$$ (1)

$$g=a+m+\epsilon_{g}$$ (2)

$$m∼N(0,\Sigma_{m})$$ (3)

$$a∼N(1,\Sigma_{a})$$ (4)

$$\epsilon_{r,g}∼N(0,\sigma_{r,g}^{2}I)$$ (5)

Where **1** denotes a vector of 1s, N(•,•) denotes a multivariate Gaussian distribution, and *σ*^*2*^_*r*_ and *σ*^*2*^_*g*_ denote the variance of the additive Gaussian noise in the red and green channels, respectively. The latent time series of the motion artifact ***m*** and neural activity ***a*** are both governed by Gaussian process priors, with prior covariances ***Σ***_***a***_ and ***Σ***_***m***_. The prior covariances ***Σ***_***a***_ and ***Σ***_***m***_ are each parameterized by a pair of hyperparameters, (*σ*^*2*^_*a*_, *τ*_*a*_) and (*σ*^*2*^_*m*_, *τ*_*m*_), where *σ*^*2*^ denotes the prior variance or amplitude, and *τ* denotes the temporal length scale, controlling the degree of smoothness. We use a stationary radial basis function (RBF) covariance function, such that the covariance between two time points depends only on the temporal separation between them:

$$\Sigma_{m_{\left(t,t^{\prime }\right)}}=\sigma_{m}^{2}exp\left(-\frac{{\left(t-t^{\prime }\right)}^{2}}{2\tau_{m}^{2}}\right)$$ (6)

$$\Sigma_{a_{\left(t,t^{\prime }\right)}}=\sigma_{a}^{2}exp\left(-\frac{{\left(t-t^{\prime }\right)}^{2}}{2\tau_{a}^{2}}\right)$$ (7)

Given this model structure, data ***r*** and ***g***, parameters ***a*** and ***m***, and hyperparameters *θ* (*σ*^*2*^_*a*_, *τ*_*a*_, *σ*^*2*^_*m*_, *τ*_*m*_, *σ*^*2*^_*r*_, *σ*^*2*^_*g*_), we fit the model in two steps. We first optimize the hyperparameters *θ* by maximizing the marginal likelihood *p*(***r***, ***g***|*θ*). We then compute the *maximum a posteriori* (MAP) estimates for ***a*** and ***m*** given the optimized hyperparameters $\hat{\theta }$ by maximizing the posterior *p*(***a***,***m***|***r***,***g***, $\hat{\theta }$).

The marginal likelihood is obtained by integrating the joint distribution of data and latents over ***a*** and ***m***, which can be computed in closed form:

$$\begin{array}{l} p(r,g|\theta )=\int p(r,g,a,m|\theta )da\;dm\\ \;=N\left([\begin{array}{c} 1\\ 1 \end{array}],[\begin{array}{cc} \Sigma_{m}+\sigma_{r}^{2}I & \Sigma_{m}\\ \Sigma_{m} & \Sigma_{a}+\Sigma_{m}+\sigma_{g}^{2}I \end{array}]\right) \end{array}$$ (8)

This quantity is the likelihood function for the hyperparameters, which can be optimized numerically to provide a maximum (marginal) likelihood estimate:

$$\hat{\theta }={argmax}_{\theta }\;p(r,g|\theta ).$$ (9)

By Bayes Rule the posterior $p(a,m|r,g,\hat{\theta })$ is proportional to the product of the likelihood function for ***a*** and ***m*** $p(r,g|a,m,\hat{\theta })$ and the prior $p(a,m|\hat{\theta })$:

$$p(a,m|r,g,\hat{\theta })\propto p(r,g|a,m,\hat{\theta })p(a,m|\hat{\theta })=N\left([\begin{array}{c} 1+m\\ a+m \end{array}],[\begin{array}{cc} \sigma_{r}^{2}I & 0\\ 0 & \sigma_{g}^{2}I \end{array}]\right)N([\begin{array}{c} 1\\ 0 \end{array}],[\begin{array}{cc} \Sigma_{a} & 0\\ 0 & \Sigma_{m} \end{array}]).$$ (10)

For this model, where prior and likelihood are both Gaussian, the posterior distribution is also Gaussian, with mean and covariance that can be expressed in closed form:

$$p(a,m|r,g,\hat{\theta })=N([\begin{array}{c} \hat{a}\\ \hat{m} \end{array}],\Lambda ),$$ (11)

where the covariance **Λ** and mean $\left[\begin{array}{c} \hat{a}\\ \hat{m} \end{array}\right]$, which corresponds to the MAP estimates for $\hat{a}$ and $\hat{m}$ are given by:

$$\Lambda ={\left(B^{⊺}C_{n}^{-1}B+C_{x}^{-1}\right)}^{-1},$$ (12)

$$\left[\begin{array}{c} \hat{a}\\ \hat{m} \end{array}\right]=\Lambda B^{⊺}C_{n}^{-1}\left[\begin{array}{c} r-1\\ g-1 \end{array}\right]+[\begin{array}{c} 1\\ 0 \end{array}],$$ (13)

$$\mathrm{with}\;B=\left[\begin{array}{cc} 0 & I\\ I & I \end{array}\right],C_{n}=\left[\begin{array}{cc} \sigma_{r}^{2}I & 0\\ 0 & \sigma_{g}^{2}I \end{array}\right],C_{x}=[\begin{array}{cc} \Sigma_{a} & 0\\ 0 & \Sigma_{m} \end{array}].$$ (14)

Note that our software implementation of TMAC relies on a Fourier-domain representation which diagonalizes the prior covariances, substantially reducing computation time [35].

### Model validation

In order to demonstrate that our model correctly infers ground truth activity and variances, we used TMAC to generate a synthetic dataset (**Fig 2B** top) with 5000 time points and a correlation time scale which roughly matches experimental datasets from **Fig 1**, such that 6 time points approximately corresponds to one second. We then used TMAC to infer both the activity and motion artifact from this synthetic dataset (**Fig 2B** middle and bottom). We find a good correspondence between the inferred activity from TMAC and the true activity (**Fig 2C**). The model also accurately infers the hyperparameters of the model (**Fig 2D**).

### Decoding Behavior

We next sought to evaluate TMAC on experimentally acquired neural population calcium imaging recordings in moving worms from [11]. As in [11] the data was preprocessed to remove missing values by linearly interpolating over time, and photobleaching was corrected by separately dividing green and red channel fluorescence by an exponential fit to the fluorescence with the decay time constant shared between neurons. Datasets with a high number of missing values were excluded (**S1 Table**).

**Fig 3:**
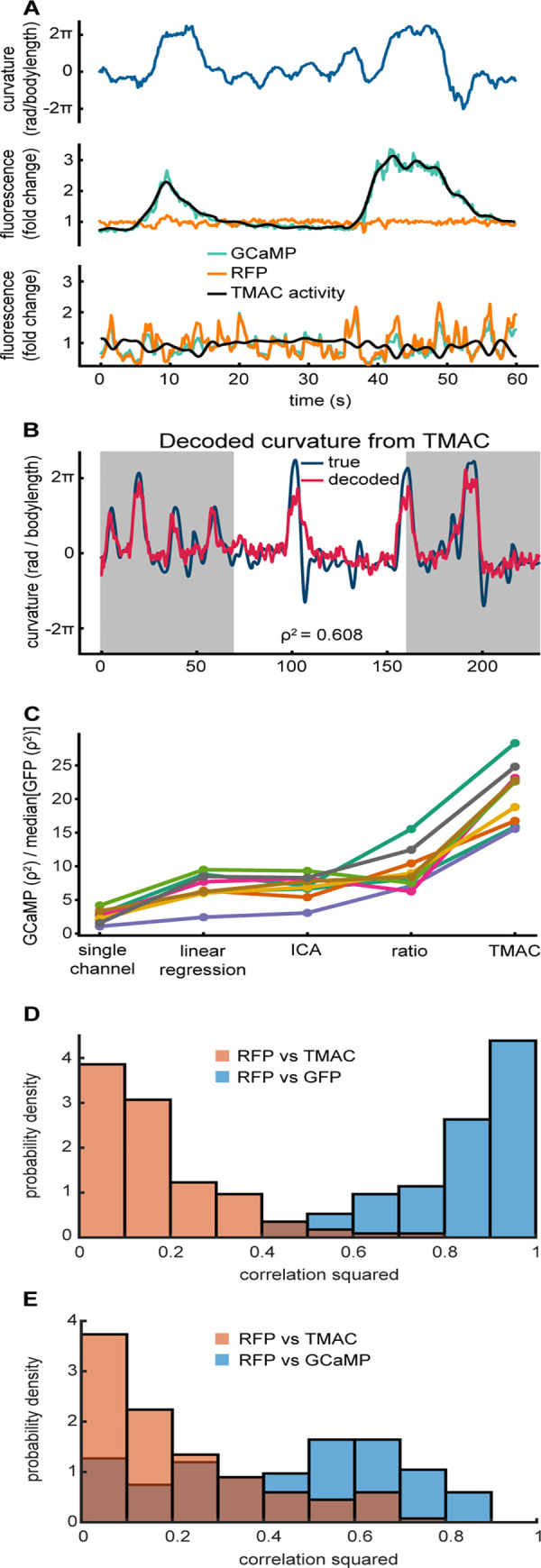
TMAC reduces decodable motion artifacts in experimental data. A) *Top*, animal body curvature over time. *Middle*, GCaMP and RFP fluorescence from a neuron that TMAC estimates to have high signal to noise, recorded from a behaving worm. *Bottom*, GCaMP and RFP fluorescence from a different neuron in that same recording that TMAC estimates to have large motion artifacts. B) Time trace of animal curvature and predicted behavior, decoded from activity inferred by TMAC in a GCaMP worm. Gray shaded regions were used to train the decoder, white region was held out and used to evaluate decoding performance. C) Ratio of decoding accuracy (ρ^2^) when decoding GCaMP divided by the median accuracy for a GFP worm across different models (**S2 Table**). D) Histogram over all neurons of correlation squared between RFP and activity inferred by TMAC from a GFP worm. RFP vs GFP data the same as in **Fig 1D and 1E**. E) Same as F but in a GCaMP worm.

The hyperparameters learned by TMAC provide a convenient method to characterize the recordings. We identified a putative high signal-to-noise neuron, by finding a neuron that the model associated with a high ratio of activity variance (*σ*^*2*^_*a*_) to motion and noise variances (*σ*^*2*^_*m*,*r*,*g*_) (**Fig 3A** middle). We also identified a putative low signal-to-noise neuron by finding a neuron that the model associated with high ratio of motion artifact (*σ*^*2*^_*m*_) to activity and noise (*σ*^*2*^_*a*,*r*,*g*_) (**Fig 3A** bottom). For completeness we also provide example fluorescence traces from neurons recorded in an immobilized worm (**S1 Fig**). In the case where TMAC estimates high signal-to-noise, TMAC predicts that a smoothed version of the green channel represents true calcium activity (**Fig 3A** middle). In the case where TMAC estimates that most fluorescent fluctuations are due to motion, TMAC deviates from the measured fluorescent intensities and returns a flatter inferred activity (**Fig 3A** bottom).

We next wanted to evaluate TMAC’s performance on removing motion artifacts. Since we lack access to the ground truth calcium activity, we considered the problem of decoding animal curvature from neural activity (**Fig 3B**). To decode the behavior of each animal we followed the same procedure from [11]. Namely, we trained a linear decoder with an L2 penalty on the weights determined using multifold cross validation. Behavior was predicted at each time point from the motion corrected activity, and all reported decoding accuracy scores are from data held out from the center 40% of the recording (**Fig 3B**).

Using decodability as a metric for motion correction performance is challenging because motion artifacts may also provide information about the target behavior. In some regimes, successfully removing motion artifacts could in principle reduce decodability rather than improve it. We therefore evaluated motion correction performance by inspecting the decodability of two types of recordings; one that had both activity-dependent and activity-independent fluorophores (GCaMP and RFP), and a control worm that had two activity-independent fluorophores (GFP and RFP), both from Hallinen et al., 2021. Although the GFP control worm contains no neural signal, the worm’s behavior can still be decoded, albeit poorly, by relying on behavior-related information in the motion artifact. We reasoned that a successful motion correction algorithm should reduce the decodability of motion artifacts while retaining the ability to decode from neural signals. We therefore define a new metric that evaluates motion correction performance as the ratio of its decodability of recordings from GCaMP animals to its decodability of recordings from GFP animals (**Fig 3C**).

Using this metric, we compare five models used for motion correction in two-channel imaging. The models are (**S2 Table**): single channel GCaMP which is just the green channel fluorescence with no motion correction, the ratio model which is the green channel divided by the red channel, a linear regression model [11], an ICA based approach [36], and TMAC. The linear regression model finds the best linear fit of the red channel to the green channel and then subtracts off that best fit from the green channel. The ICA approach performs ICA using the red and green channel as inputs and returns the independent component least correlated to the red fluorescence.

The activity fit by TMAC is ~20x more effective at predicting curvature from a GCaMP expressing worm than a control worm (**Figs 3C** and S2). We calculated the correlation between the activity inferred by TMAC and the red channel and they did not correlate strongly, suggesting that the model is successful at removing motion artifacts common to the two channels (**Fig 3D and 3E**). For completeness, we also compare each method on our synthetic dataset (**S3 Fig**).

## Discussion

Motion artifacts are prevalent in recordings of behaving animals (**Fig 1B and 1C**). These artifacts can appear as signals of interest, reducing the interpretability of the data. Here we presented TMAC, a model which infers the latent neural activity without these artifacts by leveraging information in two-channel calcium imaging recordings. We demonstrate that TMAC substantially reduces decodable motion artifacts in experimental data (**Fig 3A**) and outperforms four alternatives (**Fig 3C**).

The model we propose uses an additive interaction between the fluorescence transients caused by motion artifact and the fluorescence transients caused by neural activity. It is unclear in experimental data what the true interaction between artifact and activity is. However, even if the true interaction is not additive, TMAC will perform well under model mismatch because it can be thought of as a linear approximation of the true interaction between *a* and *m*. Consider the case where the activity and motion interact multiplicatively. Multiplicative interactions have linear components when the two variables have nonzero means. The linear component of the multiplicative interaction is clear by writing the equation for the green channel with no noise.

g=(a+1)(m+1)=am+a+m+1 (15)

We can be sure that the ***a*** and ***m*** have a mean (in this case 1), because fluorescent data is nonnegative. Furthermore, the ***am*** term itself can be approximated by ***a*** + ***m*** as long as ***a*** and ***m*** are small. If the approximation of ***a*** and ***m*** as Gaussian is valid, we can be sure that the ***am*** and higher order terms are small, because the standard deviations of ***a*** and ***m*** must be small relative to 1 to avoid negative values. For these reasons, while TMAC is a linear approximation to the true interaction between ***a*** and ***m***, it is an approximation that is highly accurate. An interesting avenue for future work would be to consider asymmetric distributions which can retain both high variability and positivity and may better approximate the true activity distribution.

In this work we demonstrated TMAC’s ability to remove decodable motion artifacts from calcium induced fluorescence. However, TMAC will remove motion artifacts in any type of two-channel imaging, so long as one channel is activity-independent and both channels share the same motion artifact component. TMAC could therefore be applied to a wide range of two-channel imaging modalities including for voltage imaging [37], fiber photometry when using an isosbestic wavelength [38,39], or two-channel two-photon imaging [40].

## Supporting information

S1 Fig TMAC inferred neural activity from an immobilized animal. A) GCaMP and RFP fluorescence from a neuron that TMAC estimates to have a high ratio of activity variance (*σ*^*2*^_*a*_) to motion and noise variances (*σ*^*2*^_*m*,*r*,*g*_), recorded from an immobilized worm. B) GCaMP and RFP fluorescence from a different neuron in that same recording that TMAC estimates to have a high ratio of motion variance (*σ*^*2*^_*m*_) to activity and noise variances (*σ*^*2*^_*a*,*r*,*g*_). Because the worm is immobilized, the motion artifacts are still small even for the highest motion variance neurons. When there is low motion artifact, TMAC estimates the activity is similar to a smoothed version of the green channel.
(PDF)

S2 Fig Motion artifact correction for GCaMP and GFP expressing animals. A) Decoding accuracy when decoding whole-body curvature from GFP recordings with different motion correction methods applied. These animals do not express an activity-dependent fluorophore so all decoding comes from motion artifacts. The mean and median of decoding of each method is listed. B) Decoding accuracy when decoding whole-body curvature from GCaMP expressing animals. The ratio of B to the median for each method in A is the value reported in **Fig 2C**. C) As in Fig 3C, but each decoding value has been divided by the mean (rather than median) decoding values from each metric in A.
(PDF)

S3 Fig Accuracy of motion correction methods on synthetic data. Each of the 5 methods for motion correction were tested on the synthetic dataset from **Fig 2**. The reported value is the distribution of correlation squared between inferred activity and true activity over instantiations of neurons. This synthetic data was generated from TMAC itself so it is unsurprising that it outperforms other methods on this dataset. The linear regression method also performs well because, like TMAC, it assumes an additive interaction between motion and activity.
(PDF)

S1 Table Data source for each figure. (DOCX)

S2 Table Models for motion artifact correction. (DOCX)

## References

[1] Grienberger C, Konnerth A. Imaging Calcium in Neurons. Neuron. 2012;73: 862–885. doi: 10.1016/j.neuron.2012.02.011 22405199

[2] Urai AE, Doiron B, Leifer AM, Churchland AK. Large-scale neural recordings call for new insights to link brain and behavior. Nature Neuroscience 2022 25:1. 2022;25: 11–19. doi: 10.1038/s41593-021-00980-9 34980926

[3] Mukamel EA, Nimmerjahn A, Schnitzer MJ. Automated Analysis of Cellular Signals from Large-Scale Calcium Imaging Data. Neuron. 2009;63: 747–760. doi: 10.1016/j.neuron.2009.08.009 19778505 PMC3282191

[4] Pnevmatikakis EA, Soudry D, Gao Y, Machado TA, Merel J, Pfau D, et al. Simultaneous Denoising, Deconvolution, and Demixing of Calcium Imaging Data. Neuron. 2016;89: 285. doi: 10.1016/j.neuron.2015.11.037 26774160 PMC4881387

[5] Nejatbakhsh A, Varol E, Yemini E, Venkatachalam V, Lin A, Samuel ADT, et al. Demixing Calcium Imaging Data in C. elegans via Deformable Non-negative Matrix Factorization. Lecture Notes in Computer Science (including subseries Lecture Notes in Artificial Intelligence and Lecture Notes in Bioinformatics). 2020;12265 LNCS: 14–24. doi: 10.1007/978-3-030-59722-1_2/FIGURES/3

[6] Friedrich J, Zhou P, Paninski L. Fast online deconvolution of calcium imaging data. PLOS Computational Biology. 2017;13: e1005423. doi: 10.1371/journal.pcbi.1005423 28291787 PMC5370160

[7] Krakauer JW, Ghazanfar AA, Gomez-Marin A, MacIver MA, Poeppel D. Neuroscience Needs Behavior: Correcting a Reductionist Bias. Neuron. 2017;93: 480–490. doi: 10.1016/j.neuron.2016.12.041 28182904

[8] Datta SR, Anderson DJ, Branson K, Perona P, Leifer A. Computational Neuroethology: A Call to Action. Neuron. 2019;104: 11–24. doi: 10.1016/j.neuron.2019.09.038 31600508 PMC6981239

[9] Dombeck DA, Khabbaz AN, Collman F, Adelman TL, Tank DW. Imaging Large-Scale Neural Activity with Cellular Resolution in Awake, Mobile Mice. Neuron. 2007;56: 43–57. doi: 10.1016/J.NEURON.2007.08.003/ATTACHMENT/A479BF36-0808-4C2B-B6EE-869BFCE0E9F0/MMC7.MOV 17920014 PMC2268027

[10] Clark DA, Gabel C v., Gabel H, Samuel ADT. Temporal activity patterns in thermosensory neurons of freely moving Caenorhabditis elegans encode spatial thermal gradients. J Neurosci. 2007;27: 6083–6090. doi: 10.1523/JNEUROSCI.1032-07.2007 17553981 PMC6672141

[11] Hallinen KM, Dempsey R, Scholz M, Yu X, Linder A, Randi F, et al. Decoding locomotion from population neural activity in moving C. Elegans. Elife. 2021;10. doi: 10.7554/ELIFE.66135 34323218 PMC8439659

[12] Lagache T, Hanson A, Pérez-Ortega JE, Fairhall A, Yuste R. EMC2: A versatile algorithm for robust tracking of calcium dynamics from individual neurons in behaving animals. bioRxiv. 2021; 2020.06.22.165696. doi: 10.1101/2020.06.22.165696 PMC8528277 34624016

[13] Yu X, Creamer MS, Randi F, Sharma AK, Linderman SW, Leifer AM. Fast deep neural correspondence for tracking and identifying neurons in C. elegans using semi-synthetic training. Elife. 2021;10. doi: 10.7554/ELIFE.66410 34259623 PMC8367385

[14] Nguyen JP, Linder AN, Plummer GS, Shaevitz JW, Leifer AM. Automatically tracking neurons in a moving and deforming brain. Dyer E, editor. PLOS Computational Biology. 2017;13: e1005517. doi: 10.1371/journal.pcbi.1005517 28545068 PMC5436637

[15] Sage D, Neumann FR, Hediger F, Gasser SM, Unser M. Automatic tracking of individual fluorescence particles: Application to the study of chromosome dynamics. IEEE Transactions on Image Processing. 2005;14: 1372–1383. doi: 10.1109/tip.2005.852787 16190472

[16] Bonneau S, Dahan M, Cohen LD. Single quantum dot tracking based on perceptual grouping using minimal paths in a spatiotemporal volume. IEEE Transactions on Image Processing. 2005;14: 1384–1395. doi: 10.1109/tip.2005.852794 16190473

[17] Chaudhary S, Lee SA, Li Y, Patel DS, Lu H. Graphical-model framework for automated annotation of cell identities in dense cellular images. Elife. 2021;10: 1–108. doi: 10.7554/eLife.60321 33625357 PMC8032398

[18] Sbalzarini IF, Koumoutsakos P. Feature point tracking and trajectory analysis for video imaging in cell biology. Journal of Structural Biology. 2005;151: 182–195. doi: 10.1016/j.jsb.2005.06.002 16043363

[19] Jaqaman K, Loerke D, Mettlen M, Kuwata H, Grinstein S, Schmid SL, et al. Robust single-particle tracking in live-cell time-lapse sequences. Nature Methods 2008 5:8. 2008;5: 695–702. doi: 10.1038/nmeth.1237 18641657 PMC2747604

[20] Chenouard N, Bloch I, Olivo-Marin JC. Multiple hypothesis tracking for cluttered biological image sequences. IEEE Transactions on Pattern Analysis and Machine Intelligence. 2013;35: 2736–2750. doi: 10.1109/TPAMI.2013.97 24051732

[21] Pnevmatikakis EA, Giovannucci A. NoRMCorre: An online algorithm for piecewise rigid motion correction of calcium imaging data. Journal of Neuroscience Methods. 2017;291: 83–94. doi: 10.1016/j.jneumeth.2017.07.031 28782629

[22] Venkatachalam V, Ji N, Wang X, Clark C, Mitchell JK, Klein M, et al. Pan-neuronal imaging in roaming Caenorhabditis elegans. Proc Natl Acad Sci U S A. 2016;113: E1082–E1088. doi: 10.1073/pnas.1507109113 26711989 PMC4776525

[23] Nguyen JP, Shipley FB, Linder AN, Plummer GS, Liu M, Setru SU, et al. Whole-brain calcium imaging with cellular resolution in freely behaving Caenorhabditis elegans. Proc Natl Acad Sci U S A. 2016;113: E1074–81. doi: 10.1073/pnas.1507110112 26712014 PMC4776509

[24] Yamamoto W, Yuste R. Whole-Body Imaging of Neural and Muscle Activity during Behavior in Hydra vulgaris: Effect of Osmolarity on Contraction Bursts. eN?euro. 2020;7: 1–13. doi: 10.1523/ENEURO.0539-19.2020 32699071 PMC7452734

[25] Vaadia RD, Li W, Voleti V, Singhania A, Hillman EMC, Grueber WB. Characterization of Proprioceptive System Dynamics in Behaving Drosophila Larvae Using High-Speed Volumetric Microscopy. Current Biology. 2019;29: 935–944.e4. doi: 10.1016/j.cub.2019.01.060 30853438 PMC6624193

[26] Karagyozov D, Mihovilovic Skanata M, Lesar A, Gershow M. Recording Neural Activity in Unrestrained Animals with Three-Dimensional Tracking Two-Photon Microscopy. Cell Reports. 2018;25: 1371–1383.e10. doi: 10.1016/j.celrep.2018.10.013 30380425 PMC6287944

[27] Kim DH, Kim J, Marques JC, Grama A, Hildebrand DGC, Gu W, et al. Pan-neuronal calcium imaging with cellular resolution in freely swimming zebrafish. Nature Methods 2017 14:11. 2017;14: 1107–1114. doi: 10.1038/nmeth.4429 28892088

[28] Cong L, Wang Z, Chai Y, Hang W, Shang C, Yang W, et al. Rapid whole brain imaging of neural activity in freely behaving larval zebrafish (Danio rerio). Elife. 2017;6. doi: 10.7554/ELIFE.28158 28930070 PMC5644961

[29] Kerr R, Lev-Ram V, Baird G, Vincent P, Tsien RY, Schafer WR. Optical imaging of calcium transients in neurons and pharyngeal muscle of C. elegans. Neuron. 2000;26: 583–594. doi: 10.1016/s0896-6273(00)81196-4 10896155

[30] Chen T-W, Wardill TJ, Sun Y, Pulver SR, Renninger SL, Baohan A, et al. Ultrasensitive fluorescent proteins for imaging neuronal activity. Nature. 2013;499: 295–300. doi: 10.1038/nature12354 23868258 PMC3777791

[31] Shipley FB, Clark CM, Alkema MJ, Leifer AM. Simultaneous optogenetic manipulation and calcium imaging in freely moving C. elegans. Front Neural Circuits. 2014;8. doi: 10.3389/fncir.2014.00028 24715856 PMC3970007

[32] Kato S, Kaplan HS, Schrödel T, Skora S, Lindsay TH, Yemini E, et al. Global Brain Dynamics Embed the Motor Command Sequence of Caenorhabditis elegans. Cell. 2015;163: 656–669. doi: 10.1016/j.cell.2015.09.034 26478179

[33] Wu Y, Wu S, Wang X, Lang C, Zhang Q, Wen Q, et al. Rapid detection and recognition of whole brain activity in a freely behaving Caenorhabditis elegans. 2021 [cited 11 Apr 2022]. doi: 10.48550/arxiv.2109.10474 PMC9584436 36215325

[34] Tai DCS, Caldwell BJ, LeGrice IJ, Hooks DA, Pullan AJ, Smaill BH. Correction of motion artifact in transmembrane voltage-sensitive fluorescent dye emission in hearts. American Journal of Physiology—Heart and Circulatory Physiology. 2004;287: 985–993. doi: 10.1152/ajpheart.00574.2003 15130885

[35] Aoi MC, Pillow JW. Scalable Bayesian inference for high-dimensional neural receptive fields. bioRxiv. 2017; 212217. doi: 10.1101/212217

[36] Scholz M, Linder AN, Randi F, Sharma AK, Yu X, Shaevitz JW, et al. Predicting natural behavior from whole-brain neural dynamics. bioRxiv. 2018; 445643. doi: 10.1101/445643

[37] Bando Y, Sakamoto M, Kim S, Ayzenshtat I, Yuste R. Comparative Evaluation of Genetically Encoded Voltage Indicators. Cell Reports. 2019;26: 802–813.e4. doi: 10.1016/j.celrep.2018.12.088 30650368 PMC7075032

[38] Lerner TN, Shilyansky C, Davidson TJ, Evans KE, Beier KT, Zalocusky KA, et al. Intact-Brain Analyses Reveal Distinct Information Carried by SNc Dopamine Subcircuits. Cell. 2015;162: 635–647. doi: 10.1016/j.cell.2015.07.014 26232229 PMC4790813

[39] Luchsinger JR, Fetterly TL, Williford KM, Salimando GJ, Doyle MA, Maldonado J, et al. Delineation of an insula-BNST circuit engaged by struggling behavior that regulates avoidance in mice. Nature Communications 2021 12:1. 2021;12: 1–18. doi: 10.1038/s41467-021-23674-z 34117229 PMC8196075

[40] Musall S, Kaufman MT, Juavinett AL, Gluf S, Churchland AK. Single-trial neural dynamics are dominated by richly varied movements. Nature Neuroscience 2019 22:10. 2019;22: 1677–1686. doi: 10.1038/s41593-019-0502-4 31551604 PMC6768091

