# Supplementary figures and images for: Correcting motion induced fluorescence artifacts in two-channel neural imaging
Source: PLoS Comput Biol. 2022 Sep 28;18(9):e1010421. doi: 10.1371/journal.pcbi.1010421 (PMC9518861; doi:10.1371/journal.pcbi.1010421)

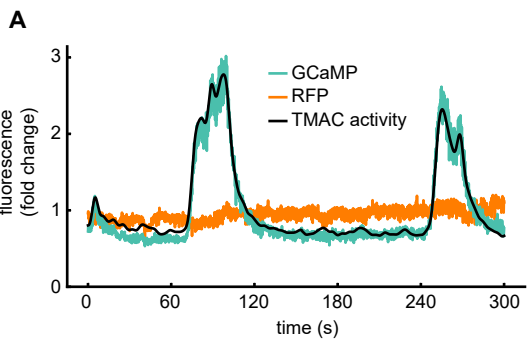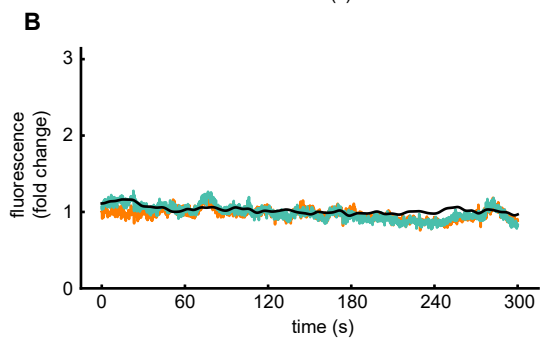

Supplement: S1 Fig — A) GCaMP and RFP fluorescence from a neuron that TMAC estimates to have a high ratio of activity variance (σ2a) to motion and noise variances (σ2m,r,g), recorded from an immobilized worm. B) GCaMP and RFP fluorescence from a different neuron in that same recording that TMAC estimates to have a high ratio of motion variance (σ2m) to activity and noise variances (σ2a,r,g). Because the worm is immobilized, the motion artifacts are still small even for the highest motion variance neurons. When there is low motion artifact, TMAC estimates the activity is similar to a smoothed version of the green channel. (PDF) [file pcbi.1010421.s001.pdf]

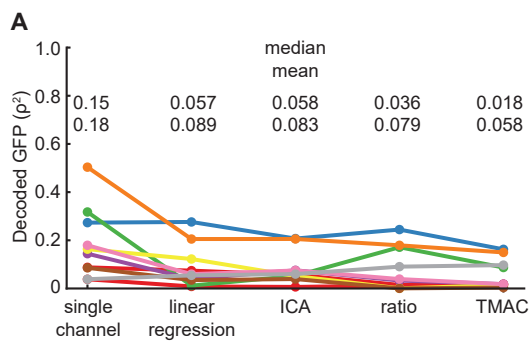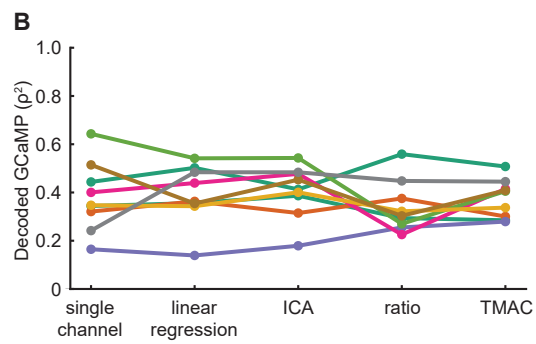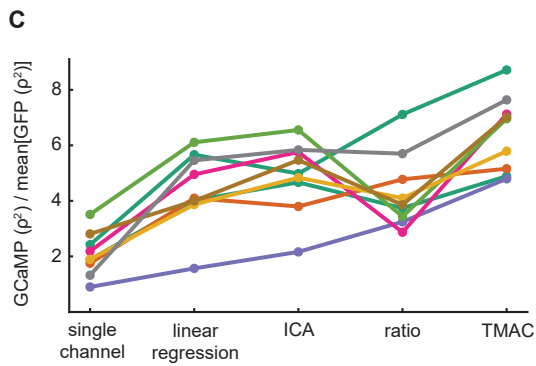

Supplement: S2 Fig — A) Decoding accuracy when decoding whole-body curvature from GFP recordings with different motion correction methods applied. These animals do not express an activity-dependent fluorophore so all decoding comes from motion artifacts. The mean and median of decoding of each method is listed. B) Decoding accuracy when decoding whole-body curvature from GCaMP expressing animals. The ratio of B to the median for each method in A is the value reported in Fig 2C. C) As in Fig 3C, but each decoding value has been divided by the mean (rather than median) decoding values from each metric in A. (PDF) [file pcbi.1010421.s002.pdf]

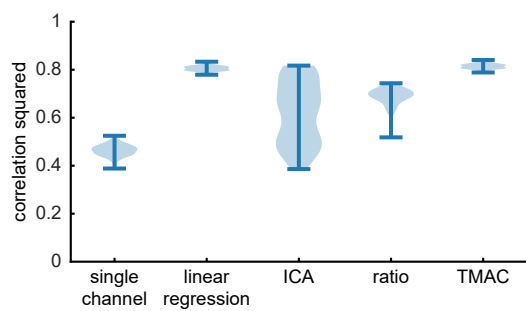

Supplement: S3 Fig — Each of the 5 methods for motion correction were tested on the synthetic dataset from Fig 2. The reported value is the distribution of correlation squared between inferred activity and true activity over instantiations of neurons. This synthetic data was generated from TMAC itself so it is unsurprising that it outperforms other methods on this dataset. The linear regression method also performs well because, like TMAC, it assumes an additive interaction between motion and activity. (PDF) [file pcbi.1010421.s003.pdf]
