# Supplementary material for: Correcting motion induced fluorescence artifacts in two-channel neural imaging
Source: PLoS Comput Biol. 2022 Sep 28;18(9):e1010421. doi: 10.1371/journal.pcbi.1010421 (PMC9518861; doi:10.1371/journal.pcbi.1010421)
Supplement: S1 Table — (DOCX) [file pcbi.1010421.s004.docx]

**S1 Table. Data source for each figure.**

|  | **Dataset from Hallinen et al. 2021 [1]** |
| --- | --- |
| **Fig. 1B** | AML18_A |
| **Fig. 1C** | AML310_A |
| **Fig. 1D** | AML18_A-C, E-K |
| **Fig. 1E** | AML310AC, AML32A-G |
| **Fig. 3AB** | AML310_A |
| **Fig. 3C** | blue: AML310_A  orange: AML310_C  red: AML32_A  yellow: AML32_B  green: AML32_C  brown: AML32_D  purple: AML32_E  gray: AML32_F  pink: AML32_G  AML18_A-C, E-K (Used to calculate median[GFP decoding]) |
| **Fig. 3D** | AML18_A-C, E-K |
| **Fig. 3E** | AML310_AC, AML32_A-G |
| **Fig. S1** | BrainScanner20201030_133026 (immobilized) |
| **Fig. S2A** | blue: AML18_A  teal: AML18_B  green: AML18_C  pink: AML18_E  purple: AML18_F  orange: AML18_G  red: AML18_H  brown: AML18_I  gray: AML18_J  yellow: AML18_K |
| **Fig. S2B** | blue: AML310_A  orange: AML310_C  red: AML32_A  yellow: AML32_B  green: AML32_C  brown: AML32_D  purple: AML32_E  gray: AML32_F  pink: AML32_G |
| **Fig. S2C** | blue: AML310_A  orange: AML310_C  red: AML32_A  yellow: AML32_B  green: AML32_C  brown: AML32_D  purple: AML32_E  gray: AML32_F  pink: AML32_G  AML18_A-C, E-K (Used to calculate mean[GFP decoding]) |

**Reference**

1. Hallinen KM, Dempsey R, Scholz M, Yu X, Linder A, Randi F, et al. Decoding locomotion from population neural activity in moving C. Elegans. Elife. 2021;10. doi:10.7554/ELIFE.66135
