# Supplementary material for: Correcting motion induced fluorescence artifacts in two-channel neural imaging
Source: PLoS Comput Biol. 2022 Sep 28;18(9):e1010421. doi: 10.1371/journal.pcbi.1010421 (PMC9518861; doi:10.1371/journal.pcbi.1010421)
Supplement: S2 Table — (DOCX) [file pcbi.1010421.s005.docx]

**S2 Table. Models for motion artifact correction.**

|  | **Description** | **Gaussian temporal filtering** | **Citation** |
| --- | --- | --- | --- |
| **Single channel** | Single channel input, no motion correction | σ = 0.83s |  |
| **Linear regression** | Finds the best linear fit of the red channel to the green channel and then subtracts off that best fit from the green channel. | σ = 0.83s | [1] |
| **ICA** | Performs ICA using the red and green channel as inputs and returns the independent component least correlated to the red fluorescence. | σ = 0.83s | [2] |
| **Ratio** | The green channel fluorescence divided by the red channel fluorescence. | σ = 0.83s | [3,4] |
| **TMAC** | Infers latent activity from a generative model of the fluorescence. Intuitively, TMAC subtracts the motion signals present in the red channel from the green channel while accounting for channel independent noise. | None |  |

**References**

1. Hallinen KM, Dempsey R, Scholz M, Yu X, Linder A, Randi F, et al. Decoding locomotion from population neural activity in moving C. Elegans. Elife. 2021;10. doi:10.7554/ELIFE.66135

2. Scholz M, Linder AN, Randi F, Sharma AK, Yu X, Shaevitz JW, et al. Predicting natural behavior from whole-brain neural dynamics. bioRxiv. 2018; 445643. doi:10.1101/445643

3. Clark DA, Gabel C v., Gabel H, Samuel ADT. Temporal activity patterns in thermosensory neurons of freely moving Caenorhabditis elegans encode spatial thermal gradients. J Neurosci. 2007;27: 6083–6090. doi:10.1523/JNEUROSCI.1032-07.2007

4. Kerr R, Lev-Ram V, Baird G, Vincent P, Tsien RY, Schafer WR. Optical imaging of calcium transients in neurons and pharyngeal muscle of C. elegans. Neuron. 2000;26: 583–594. doi:10.1016/S0896-6273(00)81196-4
